# TagGD: Fast and Accurate Software for DNA Tag Generation and Demultiplexing

**DOI:** 10.1371/journal.pone.0057521

**Published:** 2013-03-04

**Authors:** Paul Igor Costea, Joakim Lundeberg, Pelin Akan

**Affiliations:** KTH – Royal Institute of Technology, Science for Life Laboratory, School of Biotechnology, Solna, Sweden; University of Pittsburgh, United States of America

## Abstract

Multiplexing is of vital importance for utilizing the full potential of next generation sequencing technologies. We here report TagGD (DNA-based Tag Generator and Demultiplexor), a fully-customisable, fast and accurate software package that can generate thousands of barcodes satisfying user-defined constraints and can guarantee full demultiplexing accuracy. The barcodes are designed to minimise their interference with the experiment. Insertion, deletion and substitution events are considered when designing and demultiplexing barcodes. 20,000 barcodes of length 18 were designed in 5 minutes and 2 million barcoded Illumina HiSeq-like reads generated with an error rate of 2% were demultiplexed with full accuracy in 5 minutes. We believe that our software meets a central demand in the current high-throughput biology and can be utilised in any field with ample sample abundance. The software is available on GitHub (https://github.com/pelinakan/UBD.git).

## Introduction

Parallel processing of samples is a powerful method to perform multiple experiments in time and cost-effective manner. Next generation sequencing technologies provide the necessary platform to achieve this [1], [2]. Given the current developments in sequencing platforms, the scale of multiplexing will grow requiring larger numbers of barcodes due to high sequence throughput [3].

Generating unique barcodes is challenging in a number of ways. Firstly, barcodes should conform to particular constraints such as length, GC-content, cross-hybridisation with experimental sequences, absence of particular restriction enzymes to minimise their interference with the actual experiment. Secondly, they should be unique enough to allow correct assignment of sequences to their original samples. This can be problematic since there can be errors introduced in the barcodes during both their synthesis and sequencing, resulting in non-unique barcodes. Using longer barcodes could partially solve this issue however they could hamper the sequencing space for the actual sample. Therefore it is imperative to design sufficient number of barcodes with optimal length and maximal distance to each other.

A framework developed by Xu. al. (DeLOB) can generate large numbers of barcodes that can then be printed on microarrays. However uniqueness of barcodes is based on their cross-hybridization and no demultiplexing step is implemented [4]. There are a number of available software tools to design and demultiplex DNA barcodes but generation of longer ones (>15mer) is not feasible using these tools [5], [6]. A recent publication provides a DNA barcode design framework based on Hamming codes, however the design is limited since the sequence uniqueness is determined only by base-changes (Hamming distance) and does not take into account insertion and deletion events [7]. It is possible to design barcodes that are capable of detecting and correcting errors for optimal demultiplexing [5]. A resource is available to generate error-correcting binary codes up to length 30 (corresponding to 15mer DNA barcodes) after which it is challenging to discover such optimal codes [8]. Such methods may not provide sufficient number of barcodes especially after applying user-specified constraints.

We here implemented a software package for designing large numbers of barcodes of virtually any length that can be demultiplexed with full accuracy. We employed Levenshtein distance to discriminate between the barcodes [9] since insertions and deletions also occur in DNA synthesis and sequencing steps [10], [11].

TagGD is easy-to-use, fully customizable, memory-efficient, fast software package and can also be run on a desktop computer. The user needs to specify the number and length of the barcode and the uniqueness based on the combined estimated error rate of the platforms that synthesise and sequence the barcodes including the error modality of the platform. Then the software provides the user the unique barcodes that will give the maximal accuracy within the allowed error rate. If the number of barcodes is not sufficient, the user can then either increase the length of the barcode or decrease the error rate. The user can also impose constraints on the barcode sequences using the barcode configuration file. In the demultiplexing step, the software takes FASTQ files and outputs demultiplexed reads in a FASTQ file with the optional third line containing the barcode of the reads.

## Materials and Methods

### Design

First, a random barcode of the desired length and GC content is generated, then it is checked for mono, di or tri-mer repeats, complexity, self-hybridisation, presence of restriction enzyme cut motifs (if required) and edit distance to its reverse complement. It is also hybridised with primers/adapters used in the experiment to prevent its interference. Gibb's free energy and melting temperature of the self-hybridised barcodes were computed using the DNA-fold algorithm [12] and sequences with strong secondary structures are discarded. If the barcode passes these filters, it is added to a barcode pool, and its uniqueness is checked. We use Levenshtein distance to measure the distance of two barcodes in the sequence space. We also allow wildcard position at the beginning and end of the barcode to exclude barcodes that cannot be discriminated as a result of an insertion/deletion. This option is called “padding”, when it is set to 0, the set will be resistant to substitution errors but not to insertions/deletions to the same extent. The user is recommended to use the same padding option for demultiplexing that s/he used for barcode design. The generated barcodes are printed in a text file, one line containing each barcode. The edit distance distribution of pairs of barcodes is also written in another file. Almost all parameters of TagGD can be adjusted in the configuration file; an example is included within the software package.

For demultiplexing, a hash map is built, containing all the k-mers of a given length appearing in all barcodes. This map is filled by searching against with overlapping k-mers of the putative barcode sequence so that each barcode is related to the k-mers it contains. This results in reducing the search space and thus greatly improving the time necessary to identify the best hit. All barcodes that have been found through the map search will be compared to the putative sequence through a semi-global alignment that ensures full overlap with the entire barcode. Also here we employ a parallel strategy due to each barcode mapping being independent. A given number of threads are created which consume the input, and output the computed best mapping. Note that as a result of this, the order of entries is not preserved between input and output. The input and output of the demultiplexer is in FASTQ format, the details of the best-hit barcode are added to the optional third line of the output FASTQ file. TagGD supports paired-end sequencing data.

### Implementation

TagGD is implemented using C++ and parallelised using POSIX Threads and OpenMP [13], [14]. First, random DNA sequences of desired length and GC content are generated. Sequences are then filtered as specified in the previous section to ensure least interference with each other and the experiment.

**Figure 1:**
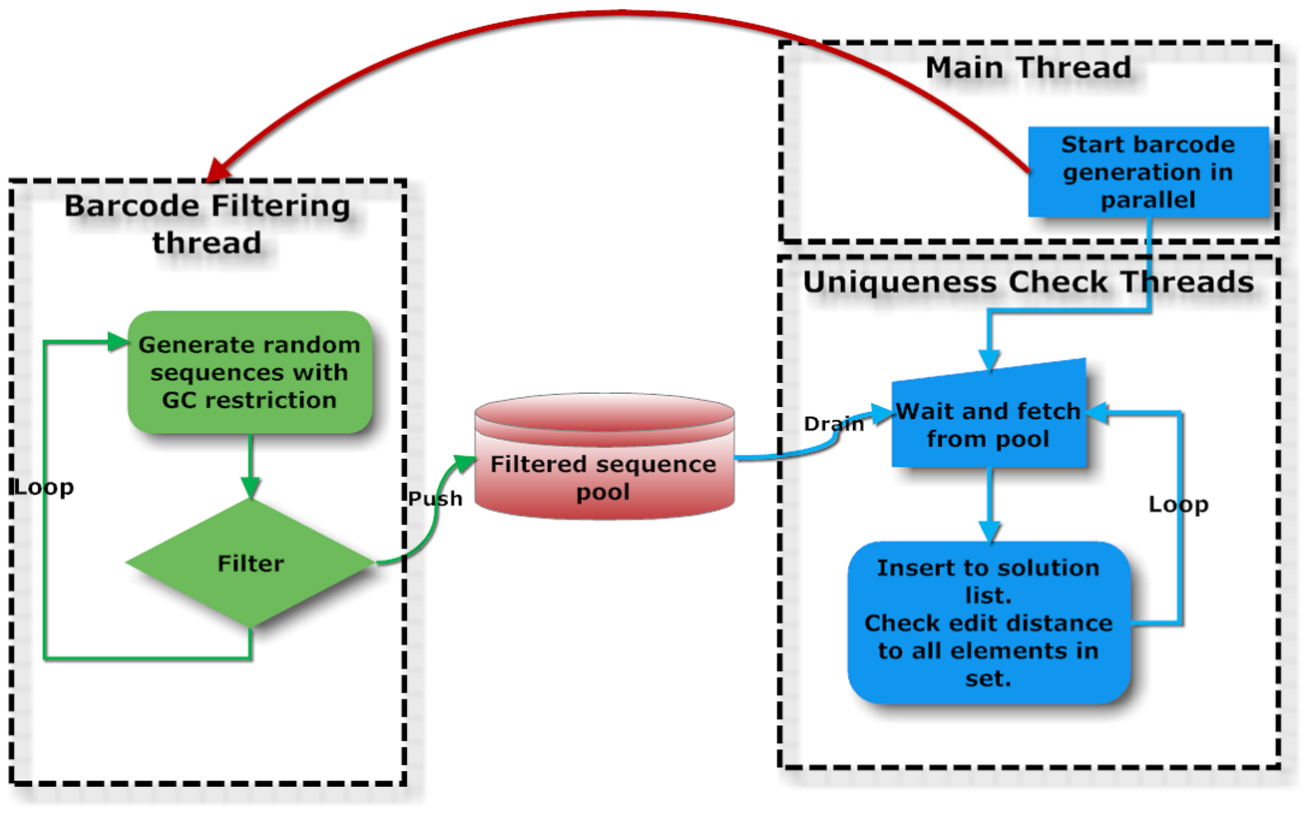
The generation of barcodes along with all the filtering steps are performed concurrently with the execution of the main application. Each barcode that passes filtering is added to a pool and generation of more barcodes continues. This pool is in time drained by the main application where the insertion into the solution set takes place.

Each barcode has to be unique. Because of possible experimental and sequencing errors, barcodes should be far away from each other in sequence space to prevent their false classification. Operations can be substitutions, insertions or deletions. The aim is to find a set of sequences where each has an edit distance greater than the desired threshold to the entire set. Thus, each new sequence that passes all the above filters is checked against the solution set and is accepted only if the edit distance to all previously accepted elements is greater than the imposed limit. As the two operations of filtering and insertion are independent, a worker thread is created to handle the generation of barcodes and push those to a common pool as shown in Figure 1. The insertion operation imposes a growing cost, scaling with the size of the solution set. Thus a parallelizing strategy is also employed here, where all the available threads are used to concomitantly search through non-overlapping subsets of the solution space. Table 1 lists the running times for generating different numbers of barcodes with and without padding option on.

**Table 1:** Running times for generating different numbers of barcodes of length 18 with different edit distances and padding.

Padding	Edit Distance	Number of Barcodes	Generation Time
0	3	5,000	18 seconds
0	4		19 seconds
0	3	20,000	4 minutes
0	4		5 minutes
0	3	100,000	1.5 hours
0	4		7 hours
1	3	1,000	1.4 seconds
1	4		2 seconds
1	3	5,000	16 seconds
1	4		46 seconds
1	3	10,000	1 minute
1	4		14 minutes

Benchmarking was performed on an 8 core 24 Gb machine.

## Results

### Assuring the uniqueness of the barcodes

**Figure 2:**
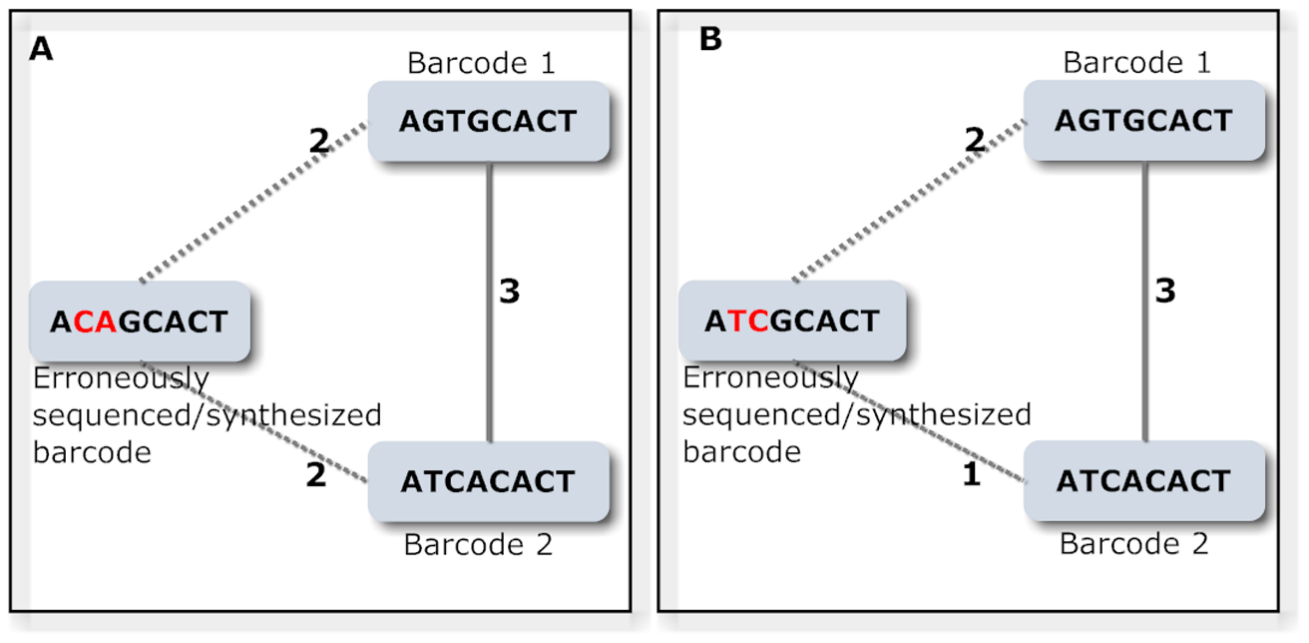
Let barcode 1 and 2 are the barcodes within the designed unique barcode set and another barcode containing errors introduced during the experiment, and the edges of the triangles represent the Levenshtein edit distance between them. A) Barcode 1 can be converted to Barcode 3 with three operations. However two errors introduced either in barcode 1 or 2 can result in a new sequence, which requires same number of operations to transform to either barcode 1 or 2. Therefore, it cannot be classified. B) Barcode 1 is incorrectly synthesised or sequenced in such a way that it now has a smaller edit distance to barcode 2, which leads to its misclassification.

Accuracy of the demultiplexing step depends on the combined error rate of the platform processing the barcodes as well as the nature of the error. We implemented TagGD to generate required number of barcodes within reasonable run times while ensuring maximal accuracy depending on the nature and the rate of the errors. An error introduced within a barcode may cause a situation that the error-containing barcode could have the same edit distance to two or more barcodes in the set, hence cannot be classified (Figure 2A). Worse case scenario is that if the error containing barcode has a smaller edit distance to another barcode than to its original, which leads to its misclassification (Figure 2B). In our experience most of the wrongly classified barcodes were due to insertion and deletion errors. Therefore we developed a strategy that deals with the problem of shifting of the barcode within the read due to insertions or deletions. To this end, we included wild card positions (padding) at the start and end of the barcode, resulting in a semi-global alignment strategy when calculating its distance to another barcode. This step ensures exclusion of barcodes that can be converted to another barcode in the set due to positional errors, insertions and deletions. Additionally, to eliminate cases where an error-containing barcode lie in equal distance to two or more barcodes, we recommend setting the minimal edit distance at least more than twice the number of bases expected to be erroneous based on the estimated error rate (Equation 1).
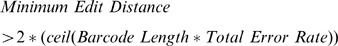
(1)


If most of the errors are expected to be due to substitutions then there is no need to design the barcodes with the padding option on so this can be set to zero. This will ensure to give the user the maximal number of barcodes within the shortest running time with full accuracy as long as equation 1 is satisfied. In other words, the barcodes can have errors as half the number of minimum edit distance in the set and can still be demultiplexed with 100% accuracy (Table 2).

**Table 2:** 20,000 unique barcodes of length 18 were generated with no padding option.

k-mer Length	Percentage of reads that are wrongly classified	Reads that cannot be uniquely classified	Running time (seconds)
9	0.27% (537)	1.7% (3423)	1.5
8	0.14% (276)	0.38% (760)	2.1
7	0.11% (223)	0.13% (267)	5.9
6	0% (0)	0% (0)	27.8

Then 200,000 barcoded reads of length 18 are generated and errors are introduced with 2% probability, only substitutions errors are allowed. With k-mer length of 6, all reads can be demultiplexed correctly in less than half a minute. Benchmarking is performed in an 8-core machine with 24 Gb memory.

However if the user expects insertion and deletion events, then the padding option must be used and the padding should be equal to the expected number of insertion or deletion events within the read. To ensure full accuracy in the demultiplexing step, equation 1 must still hold but this time errors can be substitutions, insertions or deletions.

### Demultiplexing of the barcodes

Systematic errors, in either the experiment or the sequencing can cause substitutions, insertions or deletions in the sequence. This necessitates an extra step for correctly identifying the sequenced barcode. We have implemented a semi-global aligner, in line with the edit distance definition used in generating the barcodes. It ensures the best match of a given sequence to the set of barcodes. The alignment is quality aware and will discriminate between two equally good mappings (from an edit distance perspective) based on the quality of the bases.

False negative rate of the demultiplexing depends on the k-mer length. To achieve full accuracy, the k-mer size must be set such that there will be at least one window of size k not containing an erroneous base (Equation 2). However given the low probability of having all the expected erroneous bases within the barcode, the k-mer size can be increased at a low cost to the accuracy while reducing the running time. To assess the effect of the k-mer length on accuracy, we have designed 10,000 and 20,000 barcodes of length 18 (with or without padding respectively) with a minimum edit distance of 4. We used the default settings (GC content between 45–65%, homopolymer limit 4, self hybridisation temperature 50°C). On these we simulated 200,000 reads with an error rate of 2%. Tables 2 and 3 shows the running times for demultiplexing as well as the specificity relation to k-mer length. The running times scales linearly with the number of reads demultiplexed.
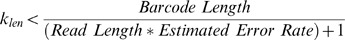
(2)


**Table 3:** 10,000 unique barcodes of length 18 were generated with padding option set to 1.

	Indexes are mapped back using positional inaccuracy set to 1			Indexes are mapped back with no positional inaccuracy		
k-mer Length	% of reads that are wrongly classified	% of reads that cannot be uniquely classified	Running time (seconds)	% of reads that are wrongly classified	% of reads that cannot be uniquely classified	Running time (seconds)
9	0.26% (525)	1.64% (3280)	2	0.13% (256)	1.84% (3684)	1.8
8	0.13% (266)	0.33% (659)	2.7	0.07% (134)	0.49% (981)	1.6
7	0.05% (105)	0.05% (96)	6.9	0.06% (123)	0.20% (400)	3.7
6	0% (0)	0% (0)	27.3	0% (7)	0% (8)	15.5

Then 200,000 barcoded reads of length 18 are generated and errors are introduced with 2% probability, substitutions, insertions and deletions are allowed. With k-mer length of 6, all reads can be demultiplexed correctly in less than half a minute, if the user uses positional inaccuracy set to 1 (equal to the padding option used in designing the barcodes). Benchmarking is performed in an 8-core machine with 24 Gb memory.

The user must provide the starting position of the barcode within the read. We allow for some variation of this site (positional error) considering that insertions or deletions may occur before the barcode location. When setting the positional error during demultiplexing, the user should consider the time cost of this (Table 3) as well as the design step padding. For example, designing with padding zero results in a fully global alignment, which over-estimates the edit distances between barcodes. We suggest the user to set the positional inaccuracy to the padding used in design stage for maximal demultiplexing accuracy. Also note that TagGD supports demultiplexing reads in which the position of the barcode is unknown, by setting the positional error to -1. This will be considerably slower and will result in less accuracy.

## Discussion

TagGD is can be downloaded from Github repository and it can be run on Linux and Windows operating systems. We provide user instructions and sample input files to ease to usage of the package (Supplementary Information). We also provide a set of scripts to test the accuracy and speed of the TagGD.

TagGD can generate large numbers of unique DNA sequences that can be used for various biological applications. We believe that TagGD can be used for any given application that requires indexing of sequences and it is implemented in a flexible way so that it can be adapted for various lengths, sequence content as well as their interference with sequences used in the experiments can be also checked against the index sequences. One may consider sequencing a large number of single cells where each cell is barcoded to provide the necessary transcriptome resolution or generating barcodes for the recently developed massively parallel gene reporter assay [15]. We also provide the algorithm to map back the index sequences with full accuracy as long as the actual error rate of the experiment does not exceed the estimated error rate used for designing the barcodes.

Barcode design unit of TagGD can be adapted to design multiplex PCR primers or microarray probes for a given genome. This would require replacing the random generation of barcodes step with given sequences from the genome of interest. Additionally demultiplexing unit of TagGD can be adapted to retrieve barcodes for multiplexed sequencing experiments to improve accuracy and speed of sample identity recovery.
